# Grant Application Review: The Case of Transparency

**DOI:** 10.1371/journal.pbio.1002010

**Published:** 2014-12-02

**Authors:** David Gurwitz, Elena Milanesi, Thomas Koenig

**Affiliations:** 1 Department of Human Molecular Genetics and Biochemistry, Sackler Faculty of Medicine, Tel Aviv University, Tel Aviv, Israel; 2 Sagol School of Neuroscience, Tel Aviv University, Tel Aviv, Israel; 3 Department of Political Science, Institute for Advanced Studies, Vienna, Austria; King's College London, United Kingdom

## Abstract

Grant Application Review: The Case of Transparency Public funding agencies should be more transparent in awarding research grants to allow researchers and the public better insight into decision making.

## The Legitimacy of Peer Reviewing

When it comes to the distribution of scarce funding resources for research in the biosciences, peer review has long been the undisputed champion of decision making. In recent years, its importance has only been enhanced, as many funding agencies increasingly distribute research funds through competitive instruments, and acquiring research funding is more important than ever for success in all branches of science [1],[2]. Today, peer reviewing has gained as much legitimacy in the scientific world as well as among lay public. Directed at the scientific community, it stands for fairness and objectivity in the distribution of grants; to the general public, it guarantees awarding of public (taxpayer) funds along scientific values rather than political ones. Robustness of procedure and efficiency of distribution are the two pillars on which the legitimacy of the peer review rests.

That is not to say that everything is perfect with peer reviewing. Studies point to a series of constraints. For example, grading of grant applications was shown to substantially differ among reviewers, a problem particularly common in biosciences [3]; moreover, reviewers differ in the weight they give to research originality, methodology, and feasibility [4]. An NSF experiment [5] found that grant reviewers who evaluated shorter, anonymized proposals selected a substantially different set of projects for funding than those chosen by reviewers presented with standard, full-length versions of the same proposals. Another study indicates that projects with high marks in the review process produce just as many publications (and citations) as projects with low marks [6]. And in a 2010 survey of grant reviewers, 85% felt they had not been sufficiently trained in grant review [7].

Opening up the “black box of peer reviewing” [8] allows funding agencies to take counter measures against what was spotted in those studies as weak features of their procedures, and to upgrade accompanying methods. We see, for example, that funders have lately put more emphasis on properly training the peer reviewers or on implementing more robust conflict of interest rules [9],[10]. In a nutshell, thus, funding agencies have done a remarkable job of increasing the robustness of their procedure by making it more and more elaborate.

The larger developments, such as the changing relationship between science and society, and the increased role that peer reviewing plays in the daily work of an average researcher, have not found the attention of funding agencies, though. As for the former, scientifically produced knowledge plays an extremely important role in our lives. It has become the basis of our economic growth; it has revolutionized the ways with which we perceive our body, our mind, our society. At the same time, and unlike what one would expect from solid scientific knowledge, this has not made societies more resilient or secure. On the contrary, the controversies over scientifically established knowledge have become harsher (think of climate change), and some sociologists of science call it now the age of uncertainty [11],[12].

What does that have to do with peer reviewing? A lot, actually. The ever growing competition on limited biomedical research funding means that more scientists submit applications for each call every year, further straining the peer review process. In the United States, the success rate of NIH grants combined has fallen from 34% in 2001 to 19% in 2012, while that of new targeted proposals fell from 28% to 14% during the same period [13]. Similar trends are evident across the globe; grant application success rates for many public funding agencies in 2012 were below 25% (Table 1). As success rates of funding opportunities are declining, writing and reviewing grant proposals is consuming more time of academic researchers than ever; a recent survey found that writing a new grant application for The National Health and Medical Research Council of Australia took principal investigators (PIs) 38 working days on average [14]. Considering that many academic researchers submit several grant applications every year, this means that much of the knowledge produced by and exchanged among researchers nowadays gravitates around peer review procedures, either as proposals or as reviews [15].

**Table 1:** Major public funding agencies, annual funding levels, application success rates, and published details of the assessment process.

Agency (*Country*) (last annual report)	Total annual funding (million US$)*	Success rate biomedical/life sciences	Abstract	Funding	Assessment summary	Final report
***NIH (USA) (2013)***	30100	14% (Medical research, 2012)	Yes	Yes	No	No
***NSF (USA) (2013–2014)***	7170	22% (General, 2013)	Yes	Yes	No	No
***Wellcome Trust (UK) (2012–2013)***	3945	25% (General, 2012–2013)	No	Yes	No	No
***JSPS (Japan) (2012–2013)***	3171	30.3% (General, 2012)	Yes	Yes	No	Yes
***DFG (Germany) (2013)***	3160	28.3% (Life Sciences)	Yes	No	No	No
***NSFC (China) (2011)***	2976	16.9% (Health Science, 2011) 20.9% (Life Sciences, 2011)	Yes	Yes	No	No
***ERC (European Union) (2013)***	2150	12%	Yes	Yes	No	No
***MRC (UK) (2013–2014)***	1357	21.6% (2013–2014)	Yes	Yes	No	No
***CONACyT (Mexico) (2012)***	937	NR	No	Yes	No	No
***NSERC (Canada) (2012–2013)***	933	NR for Biomedical/Life Sciences	No	Yes	No	No
***CSIC (Spain) (2013)***	894	NR	No	No	No	No
***SNF (Switzerland) (2012)***	789	50%	No	No	No	No
***BBSRC (UK) (2013–2014)***	777	27%	Yes	Yes	No	No
***NHMRC (Australia) (2013)***	748	20.5%	Yes	Yes	No	Yes
***Vetenskapsradet (Sweden) (2012)***	691	NR	Yes	Yes	No	No
***ANR (France) (2013)***	548	16.5% (General)	Yes	Yes	No	No
***Academy of Finland (Finland) (2014)***	395	17% (Health research, 2012)	Yes	Yes	No	No
***ZonMW (Netherlands) (2011)***	364	NR	Yes	Yes	No	No
***NCN (Poland) (2013)***	302	22% (Life Sciences)	No	Yes	No	No
***FWF (Austria) (2013)***	258	30.2% (General, 2012)	Yes	No	No	No
***FNRS (Belgium) (2012)***	225	36.9%-38.2% (2011)	No	No	No	No
***DFF (Denmark) (2013)***	205	23% (Medical science); 19% (General)	Yes	Yes	No	No
***RFBR (Russian Federation) (2013)***	200	*NR*	Yes	No	No	Yes
***Ministry of Health (Italy) (2012)***	171	*NR*	No	Yes	No	No
***CIRM (USA) (2013)***	163	42.8%	Yes	Yes	No	Yes
***ISF (Israel) (2014)***	136	32.8% (General)	No	Yes	No	No
***HFSP (International) (2012–2013)***	117	9%	Yes	No	No	No

*Arranged by decreasing total annual funding.

Funding levels and success rates are the latest as available on the funding agencies websites on October 10. 2014, rounded to nearest million US$ according to exchange rates on that day. NR: not reported.

With scientific knowledge production being increasingly enabled through peer review, and this knowledge often playing part in societal dynamics, procedural robustness and distributive efficiency of public research funding may lose their bite. A recent metastudy has concluded that “there is little empirical evidence on the effects of grant giving peer review” [16]. Similarly, concern about research integrity and scientific misconduct indicates that robustness alone no longer yields sufficient legitimacy for peer reviewing. Critics of the current system suggest thinking about drastic alternatives [17],[18], and the Open Science movement [19],[20] seeks a radical transformation of the decision-making procedures in science. In most of those accounts, transparency is entering center stage.

Transparency, however, can mean two very different things. It can mean that some knowledge within the peer reviewing procedure is openly accessible, while other information is kept away from the public. In such an understanding, transparency is supposed to buttress the existing two pillars of legitimacy, robustness of the procedure and efficiency of distribution. Or it can mean that transparency of knowledge is emerging as a new, third pillar of legitimacy that is indispensable for peer reviewing to retain its pivotal function. From that perspective, transparency of knowledge would have the potential of radically transforming peer reviewing. We can distinguish those two perspectives as incremental versus radical approaches towards transparency.

How do funding agencies currently apply transparency? Not surprisingly, as we will see, their perspective on that issue is almost exclusively an incremental one. Still, a comparative look at transparency policies of leading biomedical funding agencies across the world (Table 1) will help us to understand differences, deficiencies, and potential improvements. It will also guide our discussion following this empirical analysis, which we will divide first, to make concrete suggestions for improving transparency measures from an incremental perspective and second, to discuss, more speculatively, the transformative potential of transparency when applied under the radical perspective.

## Transparency Policies at Funding Agencies

What does transparency mean when it comes to peer reviewing grant applications? As different as the various public funding distribution modes may be, each peer review procedure typically involves a set of stages in which different knowledge items are involved. Those items can be categorized in four types: the knowledge regulating the procedure (funding agency rules), the knowledge that is examined (application content), the knowledge that is applied (expertise for evaluation), and the knowledge that is a result of the procedure (final assessment) (Table 2). Funding agencies can deal with those knowledge items in terms of access: open, if publicly available; restricted, if available only to a defined group of people; and closed, if not available at all; as well as timing: providing access to a knowledge item either before the evaluation procedure, during the procedure, or after the procedure. They define where to draw the line between containing knowledge and making it openly available, and when.

**Table 2:** Knowledge items.

Item	Knowledge category
**Description of evaluation procedure**	Rules
**Evaluation details (questions to the reviewers)**	Rules
**Names of applicants**	Content
**Names of funded PIs**	Content
**Proposals submitted to call**	Content
**Proposals/Lay Abstracts of funded projects**	Content
**Names of reviewers (at minimum, in aggregated form)**	Expertise
**Review summaries of all proposals**	Expertise
**Review summaries of funded proposals**	Expertise
**Funding budget granted to projects**	Produce
**Call success rate**	Produce
**Ranking of proposals**	Produce
**Final report (at minimum, the Final Report Abstract)**	Produce

For example, describing the basic features of the procedure on the website of the funding agency before the submission deadline of a call means that the agency treats this particular feature of information in an open way. The way one funding agency deals with the knowledge items in terms of access and timing constitutes its transparency policy. In order to assess different takes for the above knowledge items, we carefully examined the websites of 27 major public funding agencies around the world [20],[21]; (Table 1). To the best of our knowledge, such a comparative global survey has not been previously reported (a small survey of ten United Kingdom public agencies was recently reported [22]. The results of our survey tell us more about policies of individual public research funding agencies; they also allow us to make some comparison across the board and to identify best practice policies.

Unsurprisingly, transparency policies are very similar when it comes to imparting the general rules of the grant application and evaluation procedure. Similarly, the principles of the peer review procedure are made available openly, an issue coordinated also by the recently installed Global Research Council [23]. Once the peer review process is concluded, and the funding decision is made, most surveyed funding agencies publish on their website a list of projects selected for funding for each call, along with names of PIs and their affiliations and amount of funding, as well as the overall success rate of the call. Eighteen of the 27 surveyed public funding agencies also publish the scientific abstracts of successful applications (Table 1).

Other items are generally restricted by nearly all surveyed agencies. The final assessment of grants (either the result from discussion of individual reviews by the scientific review panel together with the results of the evaluation, or, where no review panel exists, simply the synopsis of the individual reviews) is sent by most agencies directly to the applicant. None of the surveyed funding bodies publishes the final assessment of funded proposals; albeit, one of them, the California Institute for Regenerative Medicine (CIRM; Table 1) publishes a “Statement of Benefit to California” as well as annual progress reports written by the authors (for example see: http://www.cirm.ca.gov/our-funding/application-reviews-rfagenomicsgc1r-06673). Adopting a common policy of publishing the final assessments would allow unsuccessful applicants the opportunity to see whether their ambitions are vindicated and, potentially, to improve their proposal based on the feedback. It will also allow the public insight into reasons for selecting certain proposals for funding.

Openly published, but only after the review procedure has been concluded, are some of the items belonging to the expertise category. Only one of the surveyed agencies, the Swedish Research Council (Vetenskapsradet), discloses the names of the reviewers before the evaluation procedure of a given call. All other funding agencies surveyed do not disclose the names of the reviewers beforehand; apparently such policy intends to block attempts by tentative applicants to manipulate the peers. Similar to the practice of many scientific journals of publishing a list of their manuscript reviewers at the end of each year (for example, [24]), some funding bodies, such as the European Research Council (ERC), publish the names of the reviewers once the review process is over. While most public grant agencies require PIs to submit final reports after the project is concluded, only four of the 27 surveyed agencies currently publish them on their websites (Table 1). Not published at all are the names of unsuccessful applicants and their proposals.

## Incremental Perspective: How to Improve Effectiveness and Robustness Further through Transparency

Our survey provides only a narrow picture, but it makes very clear that all funding agencies at their core employ a transparency policy that is primarily concentrated on providing more information about the rules of the game, and results of the procedure, while they contain scientific knowledge that is genuinely fed into the process (and is paid for by public money, as reviewers and panel members are often paid for their work), as well as knowledge that is applied to assess that content. Transparency, hence, is primarily a means to increase the robustness of the procedure and (to a lesser extent) pinpoint the efficiency of distribution. That is probably why funding agencies are rather transparent when it comes to knowledge items related to rules and procedures, but reluctant when it comes to items related to content and expertise categories.

It is important to reflect the thinking of funding agencies behind that specific pattern of regulations towards the different knowledge items involved in their peer reviewing. Apparently, it goes like this: as distributors of public funds, they need to make sure that their funding decision making is fair and objective—that it is fairly accessible by everyone eligible, that it is conducted under comprehensive conditions (without favoring someone), and that the funds are used in an accountable manner. At the same time, funders assume that for certain knowledge involved in the process (most notably, the proposals and the reviews), researchers may prefer to not be in public domain until they have published the resulting findings; as conventional wisdom goes, their unrestrained dissemination would conflict with researchers' interests of keeping their research ideas, novel technologies, and preliminary findings confidential until their publication in scientific journals. In addition, open access to individual grant review reports may damage reviewers and discourage honest review.

The apparent thinking here reflects the current system of academic research by and large. New scientific knowledge is mostly produced in a competitive mode between PIs. Funding for research is based on a meritocratic system, and peer reviewing is an essential instrument in establishing what Robert Merton once called the “stratification system of science” [25]. Accordingly, in instances where information provided is restricted (as in the case of the reviews) or delayed (as in the case of the review panel members), funders apparently intend to protect the reviewers and the scientific panel members either from interference (applicants attempting to manipulate them) or from criticism (applicants criticizing them for unjust assessment). When it comes to the unsuccessful applicants and their proposals, the general assumption is that they are better off by being shielded from the public, as well as their peers, because of risk to their reputation.

Let us follow for a moment this prevalent logic. Even from the incremental perspective, we can think of at least three measures that agencies across the world could implement quickly and without any hesitation. All are ex post approaches and should not do any harm on the evaluation procedure at all. One concerns the publication of the members of the review panels (and cumulative list of external reviewers for a specific call); this would again enhance the robustness of the peer review system, as it highlights that the peers selected for reviewing were competent and would indicate to potential applicants that their specific field of research is well covered.

Second, funding agencies may also want to consider publishing the “Impact Statement” (or similar) that is currently included as part of a grant application by many funding bodies. Publishing these sections allows the general public insight into grant selection priorities. Better transparency of the grant funding selection process would enable researchers and the public to have a better understanding of how public money is spent on scientific research; it will contribute to improved public trust in scientific research integrity while creating a new intersection for public engagement with scientific research without being detrimental to its quality or to peer review integrity.

The third suggestion concerns the publication of the final reports of funded projects. Publishing the final reports would harm neither successful grant applicants nor peer reviewers. Why this policy has not been established more broadly can only be explained by institutional inertia. In any case, we would like to see more funding agencies adopting this policy, as this gives the public better insight into the distributive efficiency of public money expenditure (Table 3). Today, final reports are often “spun” in order to comply with the formal requirements, but are otherwise written in haste. Making them public would urge PIs to make them more comprehensive, yet clear enough for the public to appreciate the project's achievements. The PI would also have to emphasize more clearly where the conduct of the project agrees with the original proposal, and what the reasons were for taking alternative routes. Thus, overall, it would increase the quality of this type of academic publication.

**Table 3:** Recommended incremental transparency measures.

Item	Should this item be published?
**Abstracts (or lay abstracts)**	Yes
**Impact statements**	Yes
**Full proposals (funded only)**	No*
**Names of external reviewers and scientific review board members**	Yes, in aggregated form, following the selection process
**Detailed (external) reviews**	No*
**Review summaries of failed proposals**	No
**Review summaries of funded proposals**	Yes
**Ranking of proposals**	Yes, only for funded proposals
**Funding budget granted to projects**	Yes
**Call success rate**	Yes
**Final reports**	Yes

*Starred items may be considered as radical transparency measures; at this time we deem it premature to recommend their open publication by default but would welcome small-scale experimentation in this area.

## The Radical Perspective: Transformative Potential of Transparency

So far, we have followed the apparent logic of the funders and have made concrete proposals to improve the implementation of transparency from an incremental perspective. However, what if we look at it from the radical perspective, assuming that transparency of knowledge would become the third pillar of legitimacy of peer reviewing? Even though there is little empirical evidence, we would expect at least two major changes. One concerns the role peer reviewing plays in the organization of scientific research. To open up the knowledge items in the content and expertise, categories would follow a very practical consideration. With all the time invested by scientists in writing reviews that remain basically invisible, making this valuable work openly accessible would avoid “reviewers' fatigue” that funders often complain about, as reviewers would know that their laborious contribution is fully acknowledgeable. Similarly, readers might find the reviews useful when assessing the reviewed work by themselves. The effects of this would be quite transformative: instead of increasing secrecy and particularism, it would become a hub for exchange of ideas and data—just as open review is already benefitting scientific manuscripts published by journals that have adopted this policy [18],[20],[26]. That, again, would considerably impact academic hierarchies and publication practices, among many others.

The other potential change concerns the role of peer reviewing in structuring the relation between science and society. So far, it has primarily served a gatekeeping function, defining who is eligible to submit and to assess proposals. Under a new regime, that boundary work would probably not be eliminated; however, the dividing line between scientific knowledge and public participation might become more permissive, as is already happening thanks to sharing knowledge through social networks [27]. Because of its important role in the academic system, opening up peer reviewing would be the most effective lever “to engage the public in scientific issues in meaningful ways in decision-making about the innovation pathways of biosciences” [19]. The important aspect here, of course, is “in meaningful ways”—that public peer review may increase the quality of both reviews and submissions is not automatically ensured, but it is worth thinking about ways to achieve this. Again, the impact of such an approach would be sweeping, including the reception of scientific knowledge (and scientists) in the public, improving public trust in scientific research.

Admittedly, all that sounds unlikely in the context of today's realities, not only because we lack the evidence, but also because it is difficult to overcome the concerns from researchers, reviewers, and funders alike. Due to the logic prevalent in academic research, the concerns relate primarily to competition within the research community. Grant winners fear that their research ideas, as well as unpublished data included in their applications may be used by competitors if rendered publicly available. Researchers and their institutions feel uncomfortable about risking their intellectual property (IP) rights. For successful applications, the risk of a fallout of the project afterwards is considered too high by reviewers supporting it, who could be concerned for their reputation. As an interim step toward more radical transparency of grant review, we believe that openly publishing the final assessment of the scientific review panel—currently published by none of the surveyed funding agencies—is the least likely to cause concerns for reviewers and funders, while going a long way toward engaging the public and increasing their trust in fairness of the grant review process and thereby in scientific research.

But it seems as if there is an even bigger concern looming in the background, namely, that opening up the entire process to the public might somehow jeopardize its legitimacy within the scientific community. While we understand those concerns, we would like to stress that the given system of conducting research is not necessarily the only one perceivable. Under the changing conditions emphasized in the opening paragraphs, there might come a situation where peer reviewing will require a third pillar of legitimacy, and this would include taking the radical perspective into account.

Advocates often refer to Winston Churchill's bon mot about democracy and say that, similarly, peer review is “the worst form of government, except for all those other forms that have been tried from time to time” [28]. We agree with that wholeheartedly. But like democracy, peer review is a principle that requires specific arrangements in order to be effective. If funders want to retain the status of peer reviewing as the fairest method of distributing funds to researchers, they must embrace transparency more actively. The definite answers to the operational questions—which transparency measures to put in place, and how—will probably only develop over time and may also depend on regional patterns of academic culture. The debate must start here and now.
